# Glioblastoma Masquerading as a Presumed Ischemic Stroke: A Case of Delusional Jealousy and Fluctuating Psychosis

**DOI:** 10.7759/cureus.115778

**Published:** 2026-09-04

**Authors:** Maria Osório, Ana Guiomar, Pedro Macedo

**Affiliations:** 1 Psychiatry Service, Unidade Local de Saúde do Tâmega e Sousa, Penafiel, PRT

**Keywords:** cerebro-vascular accident (stroke), glioblastoma idh-wildtype, hyperactive delirium, psychotic disorder, seizure differential diagnosis

## Abstract

Psychiatric symptoms can be the presenting or dominant manifestation of an underlying brain tumor, and this phenomenon is frequently misattributed to a primary psychiatric disorder, particularly when a plausible alternative explanation, such as a recent stroke or a known alcohol use disorder, is already present. We report a case in which glioblastoma was ultimately identified as the cause of a subacute psychotic syndrome that had initially been attributed to a preceding cerebrovascular diagnosis. A 64-year-old man with a history of alcohol use disorder was admitted to Internal Medicine with a diagnosis of ischemic stroke in the left middle cerebral artery territory, with new-onset seizures at admission and a self-limited episode of hyperactive delirium. Four days after discharge, he was brought to a psychiatric emergency service with heteroaggression toward his wife, near-total insomnia, pressured speech, and delusions of infidelity and persecution. A head computed tomography scan and initial laboratory workup, including toxicology, were unremarkable, and he was admitted to Psychiatry with a presumptive diagnosis of new-onset psychotic disorder. Over 14 days, despite escalating antipsychotic therapy, he developed progressively incoherent speech, disorientation, and a fluctuating level of consciousness alternating between agitation and obnubilation. Family interviews clarified that his premorbid jealousy had been alcohol-related and transient, unlike the fixed, treatment-refractory delusion now present, and that confusion and fluctuating consciousness were new since the stroke admission. Magnetic resonance imaging revealed an infiltrative left temporo-insular and basal ganglia lesion radiologically consistent with glioblastoma, later confirmed histologically as an isocitrate dehydrogenase-wildtype, grade 4 tumor. The patient underwent subtotal resection; treatment was later limited to palliative care because of tumor progression, and he died five months afterward. This case illustrates that fluctuating consciousness, disorientation, and treatment-refractory psychosis in a patient with a recent stroke or seizure disorder should prompt reconsideration of the working diagnosis and contrast-enhanced magnetic resonance imaging, since a normal computed tomography scan does not exclude an infiltrating glioma, and neighboring organic syndromes can mimic one another both clinically and radiologically.

## Introduction

Primary brain tumors are an uncommon but consequential cause of psychiatric presentations in adulthood, such as malignant gliomas that can carry a five-year overall survival rate that does not exceed 35% [1]. Malignant gliomas comprise a heterogeneous group of aggressive, infiltrative primary central nervous system tumors of glial lineage, among which glioblastoma represents the most aggressive and clinically challenging entity. Glioblastoma, a highly aggressive, diffusely infiltrating adult-type diffuse glioma characterized by rapid progression, typically presents with headache, seizures, focal neurological deficits, or cognitive change, and standard management combines maximal safe resection with radiotherapy and temozolomide, although prognosis remains poor even with treatment [2,3]. Psychiatric symptoms, however, can precede or overshadow these more classic features. Personality change, mood disturbance, and psychosis have all been documented as the sole presenting complaint of an otherwise neurologically silent tumor, a pattern that has led such cases to be described as a clinical enigma for the treating psychiatrist [4]. In a large cohort of patients with glioblastoma followed up from diagnosis through treatment, clinically relevant psychiatric symptoms requiring psychiatric referral occurred in approximately 11% of patients, most commonly mood and behavioral disturbances followed by psychotic symptoms, with non-frontal tumor location, prior psychiatric history, postoperative delirium, antiepileptic drug use, and corticosteroid initiation identified as associated factors [5]. Causation in these patients is frequently multifactorial, reflecting a combination of direct tumor effect, peritumoral edema, seizure activity including nonconvulsive status, antiseizure and corticosteroid medication, and the psychological burden of a life-limiting diagnosis, which together can obscure the underlying organic process and delay diagnosis [6].

In this report, we describe a case of patient in whom a new psychotic syndrome, emerging in the immediate aftermath of an admission for a presumed ischemic stroke and against a background of alcohol use disorder, was ultimately traced to a rapidly infiltrating glioblastoma that had, in retrospect, very plausibly already been present and already symptomatic at the time of that index admission.

## Case presentation

The patient was a 64-year-old married man, a retired construction worker with four children, with no history of regular medical follow-up, including psychiatric care, and no relevant family history. His medical history was notable for a recent hospitalization during which he had been diagnosed with an ischemic stroke. He was transferred to the psychiatric emergency service after evaluation at the general emergency department of another hospital due to a three-day history of persistent behavioral disturbances, including new-onset physical aggression toward others, increased agitation, near-total insomnia, and pressured speech. No previous episodes with a similar clinical presentation were reported. The family described occasional episodes of agitation and verbal aggression toward his wife in the context of acute alcohol intoxication, which consistently resolved as the effects of alcohol subsided, but denied any previous episodes of physical aggression. Premorbidly, he was described by his family as a hardworking, emotionally reserved, and jealous man. The change in behavior had begun one day after discharge from an Internal Medicine ward, where he had been admitted for more than 20 days with a diagnosis of ischemic stroke in the territory of the left middle cerebral artery, complicated by inaugural epileptiform activity that led to a further diagnosis of vascular epilepsy (Figure 1). That earlier admission had been prompted by a generalized tonic-clonic seizure, preceded by a short period of headache, visual hallucinations, and stereotyped movements of the upper limbs, although the exact duration of these symptoms could not be established.

**Figure 1:**
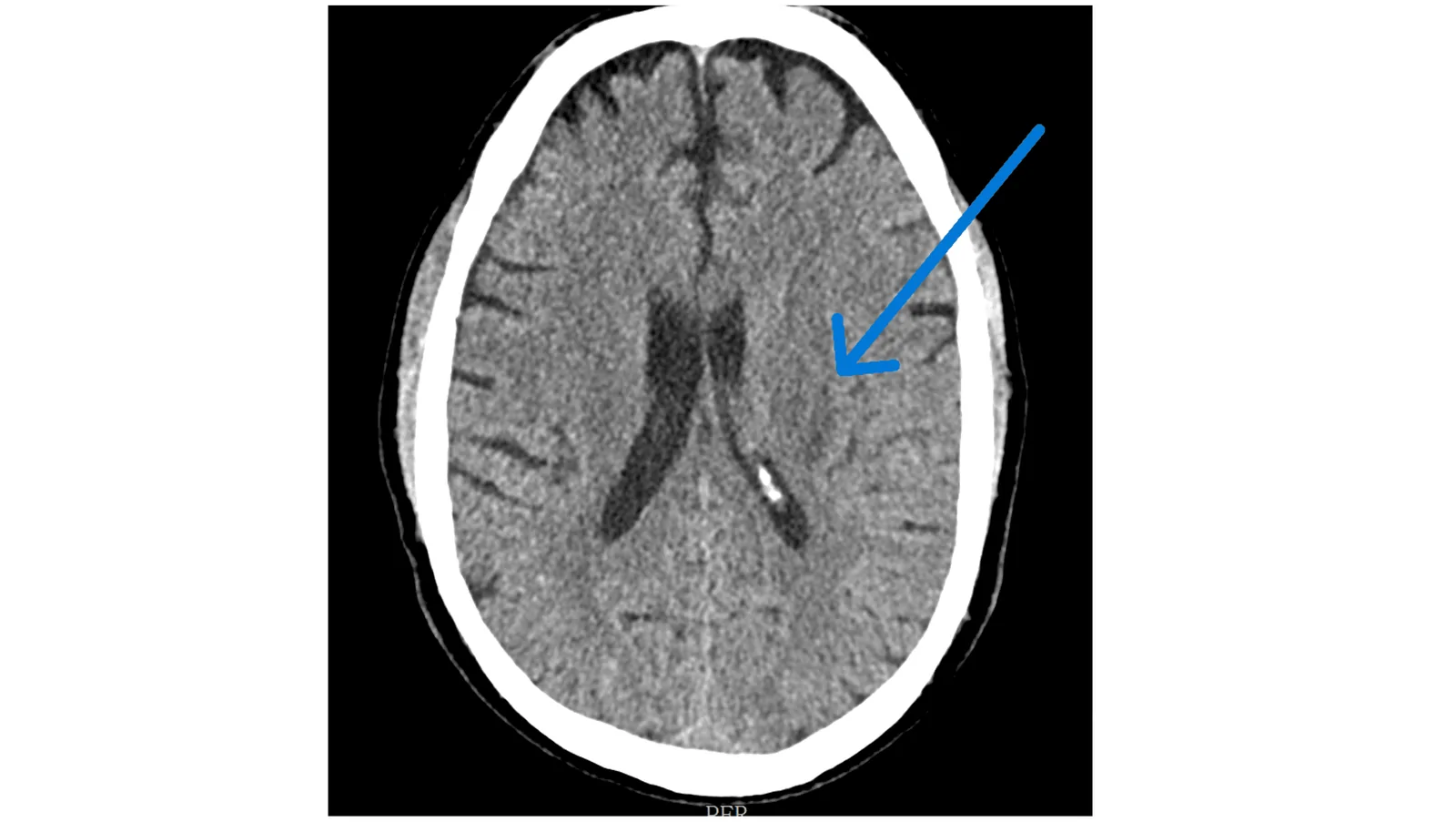
Non-contrast computed tomography scan of the brain obtained during the first admission to the Internal Medicine ward, showing a left temporal, insular, and ipsilateral lenticulocapsular hypodensity (blue arrow), consistent with the diagnosis of ischemic stroke.

His relevant history included a diagnosis of alcohol use disorder, with moderate-to-heavy daily alcohol consumption, averaging approximately one 750-mL bottle of wine per day (approximately 70 g of ethanol/day). His wife believed he had not consumed alcohol since the stroke admission but could not be certain of continued abstinence. During that admission, he developed hyperactive delirium lasting approximately 10 days, characterized by confusion, fluctuating levels of consciousness, agitation, episodes of physical aggression toward medical staff, and fragmented sleep. He was treated with haloperidol (1 mg three times daily) and oxazepam (15 mg three times daily), with complete resolution of symptoms before discharge; haloperidol was subsequently tapered and discontinued while he was still an inpatient. He was discharged with behavior described as appropriate, on oxazepam 15 mg three times daily. One day after discharge, the family noted new heteroaggression directed mainly at his wife, increased agitation, near-total insomnia, and pressured speech, along with the belief that his wife was being unfaithful. He also came to feel threatened by strangers whom he believed were in his home, reporting that he could not sleep because many people, including his wife, a neighbor, and several men, were making noise in the house and wished to harm him. At the time, he was taking oxazepam 15 mg three times daily, lacosamide 200 mg twice daily, lisinopril 5 mg once daily, acetylsalicylic acid 100 mg once daily, and rosuvastatin 20 mg once daily, corresponding to the medication regimen prescribed at discharge from the previous hospitalization. The patient was hemodynamically stable, and the general physical and neurological examinations revealed no significant abnormalities. Medical causes were excluded by the Internal Medicine team, and Neurology did not consider the presentation to be related to the recent vascular lesion. Emergency department laboratory testing showed mild anemia, leukocytosis, and thrombocytosis, while C-reactive protein, urinalysis, urine toxicology screening, blood alcohol level, and liver function tests were unremarkable (Table 1).

**Table 1:** Relevant laboratory findings obtained during the emergency department assessment

Laboratory parameter	Result	Reference range
Hemoglobin	12.5 g/dL	13.9–16.3 g/dL
White blood cell count	14.8 × 10⁹/L	4.5–11.0 × 10⁹/L
Platelet count	425 × 10⁹/L	150–400 × 10⁹/L
Glucose	101 mg/dL	76–110 mg/dL
Urea	27 mg/dL	10–50 mg/dL
Creatinine	0.91 mg/dL	0.81–1.44 mg/dL
Sodium	138 mmol/L	136–146 mmol/L
Chloride	104 mmol/L	101–109 mmol/L
Total bilirubin	0.90 mg/dL	0.3–1.2 mg/dL
Aspartate aminotransferase	31 U/L	10–37 U/L
Alanine aminotransferase	28 U/L	10–37 U/L
Gamma-glutamyl transferase	30 U/L	10–49 U/L
Alkaline phosphatase	70 U/L	30–120 U/L
Albumin	3.9 g/dL	3.5–5.2 g/dL
C-reactive protein	0.2 mg/L	<5.0 mg/L
Thyroid-stimulating hormone	0.58 µIU/mL	0.38–5.33 µIU/mL
Free thyroxine (FT4)	1.08 ng/dL	0.54–1.24 ng/dL
Free triiodothyronine (FT3)	2.98 pg/mL	2.5–4.4 pg/mL
Vitamin B12	439 pg/mL	180–914 pg/mL
Anti-Treponema pallidum antibodies	<4 U	<10 U
HIV-1/HIV-2 antibodies	Non-reactive	Non-reactive
Hepatitis B surface antigen	Non-reactive	Non-reactive
Hepatitis C antibody	Non-reactive	Non-reactive
Serum ammonia	27 µmol/L	18–72 µmol/L

A head computed tomography scan showed no post-traumatic intracranial change, no contusion or subdural hematoma, moderate leukoaraiosis, no midline shift, and a patent ventricular system and basal cisterns, with no fracture (Figure 2).

**Figure 2:**
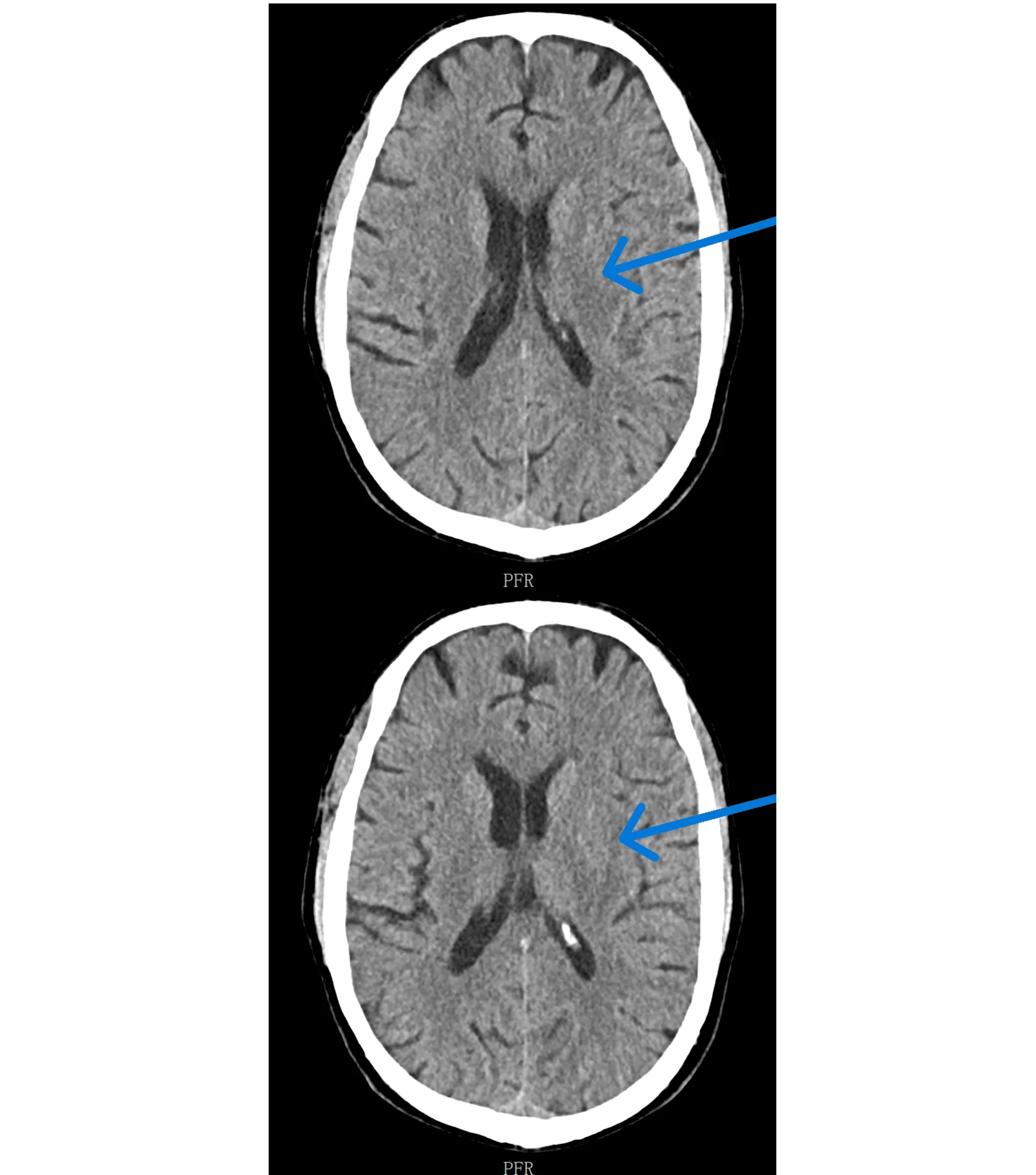
Non-contrast computed tomography scan of the brain obtained in the emergency department prior to psychiatric admission, shown at two different axial levels. The blue arrows indicate the previously identified lesion, which remained unchanged compared with the previous computed tomography scan. No new acute intracranial abnormalities were identified.

On mental status examination in the emergency department, he was alert and cooperative, partially oriented to time but oriented to place and person, with poor attention and disorganized behavior, including approaching other patients in the waiting area. Speech showed some pressure but remained interruptible, and mood was euthymic. Poorly systematized paranoid and jealous ideation was elicited, hallucinatory activity was uncertain, and insomnia had been near-total over the preceding nights. There was no active suicidal ideation. He was therefore admitted to Psychiatry for diagnostic clarification, with a presumptive diagnosis of a new-onset psychotic disorder.

During the inpatient stay, delusional jealousy persisted: he reported that his wife deleted messages from other men but that he knew everything, and he came to believe she had been unfaithful since his prior discharge, with probable auditory verbal hallucinations. Speech remained pressured and at times difficult to interrupt. Risperidone was initiated at 1 mg/day, together with thiamine 300 mg once daily, pyridoxine-cyanocobalamin supplementation (100 mg/1 mg once daily), and oxazepam (15 mg twice daily and 30 mg at bedtime), given the inability to reliably establish the timing of his last alcohol intake and, consequently, to exclude recent alcohol withdrawal. Due to the lack of clinical improvement, antipsychotic treatment was intensified with the addition of quetiapine, titrated up to 50 mg twice daily, while risperidone was adjusted to 3 mg at bedtime. Despite this change, no clinical improvement was observed, and his speech became progressively more confused and, at times, incoherent, with periods of disorientation. His level of consciousness began to fluctuate, with alternating periods of prostration, periods of relative calm without overt psychotic features, periods of recrudescent psychotic symptoms, and periods of frank agitation requiring physical and pharmacological restraint. Repeat laboratory testing during the admission was unremarkable, including a serum ammonia level of 27 micromoles per liter, within the reference range of 18-72 micromoles per liter. Family interviews with his wife clarified that he had always been a jealous man, with jealous ideation previously occurring only when intoxicated and resolving once the intoxication period passed; this was distinct from his current, unremitting delusion. She also confirmed that the periods of confused speech and fluctuating consciousness were new and had not been present before the first admission.

Given the dissociation between the clinical picture and the unremarkable emergency workup, Neurology was asked to reassess the patient for a possible ictal contribution to the presentation, and magnetic resonance imaging of the brain was requested in view of the recent stroke. Neurology raised suspicion for postictal or interictal psychosis and recommended prolonged-release sodium valproate 500 mg twice daily, with periodic monitoring of serum valproate levels. Magnetic resonance imaging performed on hospital day 14 identified an extensive infiltrative corticosubcortical lesion involving the left lateral, anterior, and medial temporal lobe, the insula, the basal ganglia, and the posterior limb of the internal capsule, with irregular and poorly defined margins. The lesion was hyperintense on T2-weighted and fluid-attenuated inversion recovery sequences, hypointense on T1-weighted sequences, and showed heterogeneous contrast enhancement in the temporal and insular regions, with a hypointense cystic-necrotic area, findings consistent with glioblastoma multiforme. The lesion effaced the left lateral ventricle and produced a 6.4 mm midline shift to the right, with mild compression of the left cerebral peduncle; there was no evidence of an acute vascular, infectious, or traumatic lesion (Figure 3).

**Figure 3:**
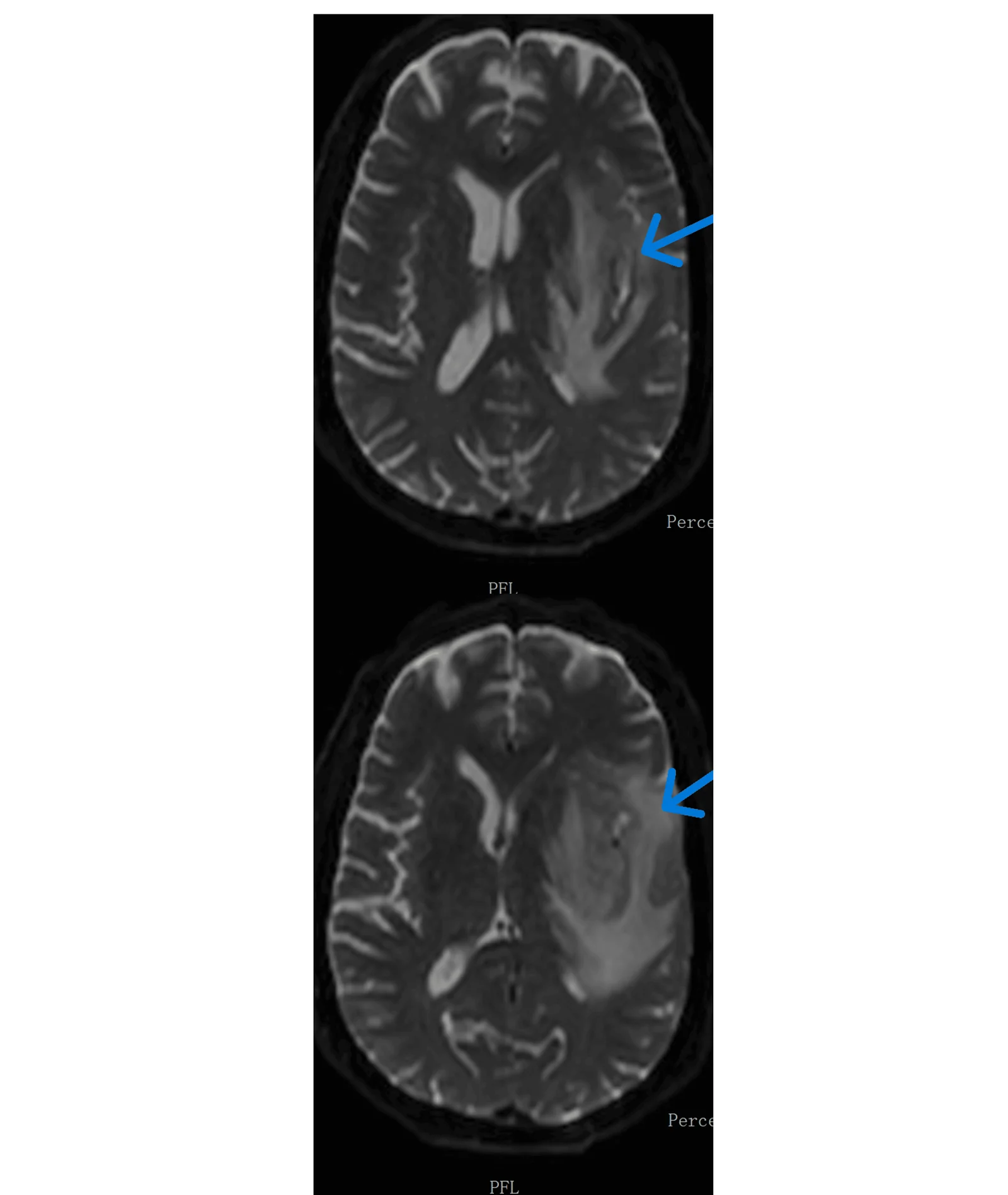
Axial T2-weighted magnetic resonance imaging of the brain obtained during psychiatric hospitalization, shown at two different axial levels. The blue arrows indicate an extensive hyperintense infiltrative corticosubcortical lesion involving the left temporal and insular regions, with extension to the basal ganglia and internal capsule. The imaging findings were consistent with glioblastoma multiforme.

Over the following 10 days, the patient's condition progressively worsened, with fluctuations between periods of obnubilation, during which he was arousable only with intense or repeated stimulation, and periods of wakefulness, during which he could direct his gaze, attempt to communicate despite unintelligible speech, follow simple commands such as tracking a finger with his eyes, and cooperate with feeding. Motor function was globally reduced; he was unable to ambulate without support, and he could not feed himself, although he cooperated when fed by others. He was transferred to the neurosurgery department of another hospital, where he was diagnosed with a left temporal glioblastoma, isocitrate dehydrogenase (IDH)-wildtype, World Health Organization (WHO) grade 4, and underwent a left anterior temporal lobectomy for subtotal resection two weeks later. Temozolomide was initiated orally at 75 mg/m² once daily concomitantly with radiotherapy. Treatment was discontinued after 15 days because of tumor progression. Following discharge from the neurosurgery department, he continued to be followed up by a multidisciplinary team comprising Neurosurgery and Oncology. Approximately two months after resection, he was readmitted to the neurosurgery inpatient unit with a one-week history of worsening generalized weakness, imbalance, right-sided limb edema, dysarthria noted by his wife, and constipation, following a fall three days earlier with impact on the right side of his body. He had no fever, nausea, or vomiting and was tolerating oral intake. At outpatient neurosurgical review shortly before this admission, he had shown severe cognitive impairment, predominantly motor aphasia with severe dysarthria, and grade 4 out of 5 right hemiparesis, precluding further formal neurological examination. He was maintained at that time on quetiapine 50 mg twice daily, risperidone 3 mg at night, valproic acid 750 mg every 12 hours, lacosamide 100 mg twice daily, and oxazepam 15 mg with an evening double dose. Repeat brain magnetic resonance imaging raised suspicion of tumor recurrence. Following multidisciplinary discussion involving Neurosurgery, Oncology, and Palliative Care, further oncological treatment was considered unfeasible given the evidence of tumor progression, and the patient was transitioned to palliative care. His psychopharmacological regimen remained unchanged at the time of transition to palliative care. He died five months later.

## Discussion

Psychiatric presentations of brain tumors

Cognitive dysfunction affects up to 90% of patients with supratentorial tumors, and frontal, temporal, and corpus callosal tumors in particular tend to produce subtle presentations, such as personality change, mood disturbance, and memory impairment, with comparatively few focal neurological signs [6]. Circuits governing executive function, emotional regulation, and social cognition appear to be a common substrate for tumor-driven delusions and aggression, and case reports have documented forensically significant new-onset offending behavior in patients whose presentation was initially diagnosed as a persistent delusional disorder [1,7]. Documented cases of glioblastoma presenting exclusively with psychiatric symptoms, without accompanying neurological signs, support a low threshold for neuroimaging whenever mental status change is atypical for a primary psychiatric illness [4]. As in our patient, causation is often multifactorial: tumor burden, peritumoral edema, seizure activity including nonconvulsive status epilepticus, corticosteroids, antiseizure medication, and the psychological burden of illness can all contribute simultaneously, so symptoms frequently cannot be attributed to a single mechanism [3,6].

Post-stroke psychosis, the alternative diagnosis initially assumed

Because our patient’s psychiatric symptoms emerged in the immediate aftermath of an admission diagnosed at the time as an ischemic stroke, post-stroke psychosis was a reasonable initial working diagnosis. A systematic review estimated the prevalence of post-stroke delusions at approximately 4.7% and hallucinations at approximately 5.1%, with a mean age at onset of 66.6 years, a male predominance, frequently delayed onset, and delusional disorder as the most common phenotype; a significant minority of affected patients had strokes that were otherwise clinically silent [8]. Lesions associated with post-stroke psychosis are typically right-sided, most often frontal, temporoparietal, or involving the right caudate, and right lateral prefrontal cortex damage, or disconnection from it, has been proposed as a common pathway to delusion formation regardless of the underlying etiology [8,9]. Approximately 3% of strokes present atypically as delirium, a delusional state, or mania mimicking a primary psychiatric illness, typically with a right-sided frontal or parietal lesion and absent or only transient neurological signs, a pattern that can plausibly delay recognition of an alternative structural cause such as a tumor [10]. In our patient, the left-sided location of both the prior stroke and the eventual glioblastoma diverges from the classic right-hemispheric localization described for post-stroke psychosis, which in retrospect was a subtle clue against a purely post-stroke mechanism, although it was not recognized as such at the time.

Delusional jealousy as an organic marker

Delusional jealousy, traditionally referred to as Othello syndrome, is characterized by a fixed and unfounded belief that one’s partner is unfaithful, maintained despite the absence of objective evidence and contrary evidence. The most localizing clinical feature in this case was the delusion of infidelity itself. In a systematic review of 73 cases of delusional jealousy, where the mean age was 58 years and there was a male predominance, 52% of cases were attributable to another medical condition, most commonly a cerebrovascular event, with 26% attributable to medications or substances and only 22% to a primary psychiatric disorder; among imaged cases, 60% had right-sided lesions and 28% had frontal lesions [11]. A separate retrospective series of 105 patients with delusional jealousy found an associated neurological disorder in 73 patients, and among the 8 patients with an identifiable structural lesion, 7 had right frontal pathology [12]. Violence has been reported in roughly a third of delusional jealousy cases, which is directly relevant to safety planning for the spouse who is the object of the delusion [11,13]. Notably, our patient's premorbid jealousy had been alcohol-related and self-limited, a pattern that is itself well described in delusional jealousy, and initially reinforced the presumption of a substance-related or primary psychiatric explanation rather than an organic one [13].

Diagnostic reasoning: computed tomography versus magnetic resonance imaging

In unselected first-episode psychosis, the yield of structural neuroimaging is comparatively low, with clinically relevant abnormalities on computed tomography estimated at 0% to 1.5%, and on magnetic resonance imaging at approximately 5.9%, as found in one meta-analysis, corresponding to a number needed to scan of 18 to detect one clinically relevant finding, which helps explain why guidelines diverge on the routine use of imaging in this population [14-16]. Our patient, however, was not an unselected case of first-episode psychosis: a recent seizure, headache, focal neurological history, hallucinations in more than one modality, and a fluctuating level of consciousness are all red flags that place a presentation outside the low-yield "new-onset psychosis" category and instead warrant investigation along seizure, stroke, and headache pathways, for which appropriateness criteria specifically favor magnetic resonance imaging with and without contrast [15]. Contrast-enhanced magnetic resonance imaging is the modality of choice whenever a tumor is suspected, and a normal computed tomography scan, as occurred at initial presentation in our patient, does not exclude an infiltrating glioma [2,3,15]. In retrospect, the "middle cerebral artery infarct with vascular epilepsy" diagnosed 20 days earlier is plausibly attributable, at least in part, to the same underlying lesion, since subacute infarction and an infiltrating glioma are well-recognized radiological mimics of one another and computed tomography is markedly less sensitive than contrast-enhanced magnetic resonance imaging for intra-axial tumor [2].

Reappraising the index presentation

In retrospect, the constellation of symptoms that prompted the first admission, namely headache, visual hallucinations, stereotyped movements of the upper limbs, and an eventual generalized tonic-clonic seizure, is at least as consistent with an evolving glioblastoma as with an acute ischemic event. Glioblastoma may mimic ischemic stroke, particularly when focal neurological deficits or seizures develop acutely, while headache, progressive symptoms, and an atypical clinical course may suggest an underlying neoplastic process. Headache from mass effect and peritumoral edema, visual hallucinations and stereotyped limb movements as manifestations of focal seizure activity, and secondary generalization to a tonic-clonic seizure are all recognized presentations of malignant glioma, and gliomas are well documented to mimic acute cerebrovascular syndromes closely enough to be misdiagnosed as such [2,10]. The subsequent psychiatric symptoms followed a similarly layered pattern of misattribution: they were initially assumed to reflect a primary psychiatric disorder, and only later, once their temporal proximity to the recent hospitalization became apparent, were they reconsidered as a possible complication of the previously diagnosed or a new cerebrovascular event, a line of reasoning that remained anchored to the original vascular diagnosis rather than questioning it outright. Because only a non-contrast head computed tomography scan was performed during the index admission, the underlying tumor went undetected, and the presentation was accepted as vascular; only when contrast-enhanced magnetic resonance imaging was eventually obtained, 14 days into the subsequent psychiatric admission, was the true nature of the lesion clarified. When a brain tumor is suspected, contrast-enhanced magnetic resonance imaging is the preferred imaging investigation, providing superior characterization of tumor location, extent, enhancement, edema, and mass effect. In patients with an atypical or evolving presumed stroke presentation, timely MRI and reassessment of the initial diagnosis may therefore reduce diagnostic delay [2,15].

Delirium mislabeled as psychosis, and the alcohol confounder

Fluctuating consciousness and disorientation, present throughout this patient's inpatient course, are the principal features that distinguish delirium from a primary psychotic disorder. Current diagnostic guidance emphasizes that late age at onset, in the absence of a personal or family history of psychotic illness, should prompt thorough assessment for a psychotic disorder due to another medical condition rather than an assumption of primary psychiatric illness [17]. The patient's alcohol use disorder functioned as a powerful diagnostic anchor throughout his course: alcohol withdrawal, alcoholic hallucinosis, Wernicke encephalopathy, and delusional jealousy are all classically associated with heavy alcohol use, and each offered a seemingly sufficient explanation for his presentation that delayed consideration of a structural cause [17]. Failure to respond to escalating antipsychotic doses should itself be treated as a diagnostic signal prompting reassessment rather than as an indication for further dose escalation; in any neuro-oncological context, new or worsening alterations in thought process should trigger investigation for a treatable underlying cause, including tumor progression [3]. Finally, nonconvulsive status epilepticus and post-ictal psychosis merit consideration as an alternative or contributory explanation for fluctuating consciousness in any patient with a recent first seizure, and electroencephalography is warranted in this setting, as illustrated by reports of subacute encephalopathy following stroke and seizure that were ultimately attributed to an ictal or peri-ictal mechanism [18].

Clinical recommendations and implications

Glioblastoma carries a poor prognosis, with a median overall survival of approximately 15 months despite treatment and a five-year survival rate below 10%. Increasing age is the strongest established demographic risk factor, while exposure to ionizing radiation and rare hereditary cancer syndromes are recognized risk factors; nevertheless, most cases occur sporadically without an identifiable predisposing factor. In patients with adequate performance status, prompt diagnosis enables timely standard-of-care management, consisting of maximal safe surgical resection followed by radiotherapy with concomitant and adjuvant temozolomide [3,6]. Antipsychotic use should generally be minimized in patients with cerebrovascular disease, given its association with increased stroke risk; when needed for agitation or safety, the lowest effective dose of a high-potency agent is preferred, with monitoring for a lowered seizure threshold and interaction with antiseizure medications [6,9]. Benzodiazepines are best avoided as first-line anxiolytics in patients with glioma because of the risk of sedation, disinhibition, and worsened cognition, whereas selective serotonin reuptake inhibitors appear safe for comorbid depression and do not appear to worsen survival [6]. Corticosteroids, frequently required perioperatively in patients with frontal or temporal tumors, can precipitate or worsen behavioral disturbance and should be anticipated as a contributing factor once initiated [5,6]. Safety planning around a delusion of infidelity is essential, given that the spouse is typically the direct target of the delusion and that violence has been reported in roughly a third of delusional jealousy cases [11]. Once the patient is medically stabilized, formal neuropsychological evaluation remains the gold standard for characterizing cognitive deficits and guiding rehabilitation, since brief bedside cognitive screens are relatively insensitive to deficits in executive function, sustained attention, and processing speed. The findings of this case have important implications for clinical practice. In older patients presenting with first-onset psychotic or behavioral symptoms, particularly in the presence of recent neurological events, diagnostic assessment should remain dynamic rather than anchored to an initial psychiatric, vascular, or substance-related explanation. An atypical or deteriorating clinical course should prompt renewed multidisciplinary evaluation and, when appropriate, more sensitive neuroimaging to exclude an underlying structural brain disorder.

## Conclusions

This case demonstrates that new-onset psychotic symptoms in an older adult can be the presenting feature of an underlying malignant brain tumor rather than a primary psychiatric illness, and that this possibility is easily obscured when a plausible alternative diagnosis, such as a recent stroke or a known alcohol use disorder, is already present. Fluctuating consciousness, disorientation, and a poor or absent response to escalating antipsychotic treatment should be treated as red flags prompting diagnostic reassessment rather than further pharmacological escalation, and a normal computed tomography scan should never be equated with structural exclusion in a patient with neurological red flags. Closer collaboration and mutual education between Psychiatry, Neurology, and Emergency Medicine, particularly around the overlapping presentations of stroke, epilepsy, and brain tumors, is essential for the timely recognition of these organic syndromes and for improving outcomes in this vulnerable patient population.

## References

[1] Lapointe S, Perry A, Butowski NA (2018). Primary brain tumours in adults. Lancet.

[2] Omuro A, DeAngelis LM (2013). Glioblastoma and other malignant gliomas: a clinical review. JAMA.

[3] (2026). NCCN Clinical Practice Guidelines in Oncology: Central Nervous System Cancers. National Comprehensive Cancer Network. https://www.nccn.org/professionals/physician_gls/pdf/cns.pdf.

[4] Moise D, Madhusoodanan S (2006). Psychiatric symptoms associated with brain tumors: a clinical enigma. CNS Spectr.

[5] Regli LK, Huijs SM, Pasmans RC (2023). Incidence of clinically relevant psychiatric symptoms during glioblastoma treatment: an exploratory study. J Neurooncol.

[6] Boele FW, Rooney AG, Grant R, Klein M (2015). Psychiatric symptoms in glioma patients: from diagnosis to management. Neuropsychiatr Dis Treat.

[7] Smith AJ, Hagen U, Brela B, Buadze A, Liebrenz M (2025). Glioblastoma and new-onset criminal behaviors in a geriatric patient: a forensic-psychiatric case report from Switzerland. Front Psychiatry.

[8] Stangeland H, Orgeta V, Bell V (2018). Poststroke psychosis: a systematic review. J Neurol Neurosurg Psychiatry.

[9] Joyce EM (2018). Organic psychosis: the pathobiology and treatment of delusions. CNS Neurosci Ther.

[10] Edlow JA, Selim MH (2011). Atypical presentations of acute cerebrovascular syndromes. Lancet Neurol.

[11] Park JH, Sarwar S, Hassett LC, Staab JP, Fipps DC (2024). Clinical characterization, course, and treatment of Othello syndrome: a case series and systematic review of the literature. J Acad Consult Liaison Psychiatry.

[12] Graff-Radford J, Whitwell JL, Geda YE, Josephs KA (2012). Clinical and imaging features of Othello's syndrome. Eur J Neurol.

[13] Seeman MV (2016). Pathological jealousy: an interactive condition. Psychiatry.

[14] Soares BP, Shih RY, Utukuri PS (2024). ACR Appropriateness Criteria® altered mental status, coma, delirium, and psychosis: 2024 update. J Am Coll Radiol.

[15] Blackman G, Neri G, Al-Doori O (2023). Prevalence of neuroradiological abnormalities in first-episode psychosis: a systematic review and meta-analysis. JAMA Psychiatry.

[16] Forbes M, Stefler D, Velakoulis D (2019). The clinical utility of structural neuroimaging in first-episode psychosis: a systematic review. Aust N Z J Psychiatry.

[17] American Psychiatric Association (2022). Diagnostic and Statistical Manual of Mental Disorders, Fifth Edition, Text Revision (DSM-5-TR).

[18] Wang T, Safadi AL, Lee EC (2022). Clinical reasoning: a 56-year-old man with unusual presentation of subacute encephalopathy and seizure. Neurology.

